# Clinical features and hypoxic marker expression of primary sinonasal and laryngeal small-cell neuroendocrine carcinoma: a small case series

**DOI:** 10.1186/1477-7819-12-199

**Published:** 2014-07-01

**Authors:** Liang Chai, Hong-Fang Ying, Ting-Ting Wu, Shui-Hong Zhou, Yang-Yang Bao, Hong-Tian Yao, Qi-Han You

**Affiliations:** 1 Department of Otolaryngology, The First Affiliated Hospital, College of Medicine, Zhejiang University, 79 Qingchun Road, Hangzhou City, Zhejiang Province 310003, China; 2 Department of Pathology, The First Affiliated Hospital, College of Medicine, Zhejiang University, 79 Qingchun Road, Hangzhou City, Zhejiang Province 310003, China

**Keywords:** Small-cell neuroendocrine carcinoma, Larynx, Nasal cavity and paranasal sinuses, Hypoxia-inducible factor-1α, Glucose transporter-1, PI3K/Akt pathway

## Abstract

**Background:**

Small-cell neuroendocrine carcinoma (SCNEC) of the head and neck is rare. The prognosis of SCNEC in the nasal cavity and larynx is poor. The aim of this study was to investigate the clinicopathological features of nasal and laryngeal SCNEC and to determine the expression of HIF-1α, GLUT-1, PI3K, and p-Akt in SCNEC.

**Methods:**

Between 2003 and 2012, 10 consecutive patients with histologically demonstrated nasal and laryngeal SCNEC were enrolled. Clinicopathological materials and follow-up data were analyzed retrospectively. Immunohistochemistry was used to detect GLUT-1, HIF-1α, PI3K, and p-Akt expression in paraffin wax-embedded tumor specimens.

**Results:**

The subjects were eight males and two females with a mean age of 60.8 (range: 53 to 71) years. Tumors were located in the maxillary sinus (n = 3) and larynx (n = 7). At last follow-up, four patients (40.0%) had local recurrence and five patients (50.0%) had developed distant metastases. Six patients died. The mean overall survival was 19.3 ± 2.1 months. Expression of GLUT-1, HIF-1α, PI3K, and p-Akt was seen in sinonasal and laryngeal SCNEC in 80 (8 out of 10), 50 (5 out of 10), 40 (4 out of 10), and 40% (4 out of 10) of cases, respectively. Expression of GLUT-1, HIF-1α, PI3K, and p-Akt was higher in sinonasal and laryngeal SCNEC than in precancerous lesions.

**Conclusions:**

Primary sinonasal and laryngeal SCNEC is rare. This paper presents 10 cases of sinonasal and laryngeal SCNEC with more common local recurrence and distant metastasis. HIF-1α, GLUT-1, PI3K, and p-Akt expression was higher in sinonasal and laryngeal SCNEC than in precancerous lesions.

## Background

Small-cell neuroendocrine carcinoma (SCNEC) of the head and neck is rare. Approximately 180 cases of the larynx and 75 cases of nasal and paranasal cavities have been reported in the English literature [1]; these were mostly in males around 50-years-old and heavy smokers [1-3]. The prognosis of SCNEC in the nasal cavity and larynx is poor. Extra-pulmonary small-cell carcinoma is usually a fatal disease, with a 13% five-year survival rate [4]. In the larynx, the two-year survival rate was 16% and the five-year rate was only 5% [5]. In the nasal cavity and sinus, the median survival time was two to three years [6]. The survival rates are similar to those for small-cell lung cancer. Unfavorable prognostic factors of SCNEC in the head and neck show a correlation with an invasion of the lamina cribrosa [7], ectopic hormone syndrome [8], recurrence, and distant metastasis [5]. However, tumor size and number of mitoses show no correlation with recurrence, metastasis, or survival [5]. Conclusive prognostic factors remain unclear because of the limited number of SCNEC cases in the head and neck. Thus, prognostic factors need further study using a greater numbers of cases of SCNEC in the head and neck.

Some studies have investigated the prognostic factors for SCNEC, looking at molecular markers, such as hypoxia-inducible factor-1α (HIF-1α).HIF-1αhas been studied in neuroendocrine carcinoma (NEC) [9,10]. Hypoxia in solid tumors has been associated with therapy resistance and poor clinical prognosis [9,10]. HIF-1α is upregulated in a wide range of solid tumors in humans, and over-expression of HIF-1α is associated with tumor aggressiveness and poor prognosis [9,10]. In small-cell lung cancer, a few studies have revealed that high levels of HIF-1α is correlation with poor survival [9,10]. GLUT-1, a major protein of cellular glucose uptake, has been studied in NEC [11]. Hypoxia promotes the chemo-radioresistance of carcinomas [12] and GLUT-1 is overexpressed in a hypoxic environment [13]. HIF-1α, a transcription factor associated with the cellular response to hypoxia [14], upregulates the expression of several hypoxia response genes, including GLUT-1. We have previously reported that there is a significant correlation between GLUT-1 and HIF-1α expression in laryngeal carcinoma [15], and there is further evidence thatthe phosphatidylinositol 3-kinase (PI3K)/protein kinase B (Akt) pathwaymay regulate HIF-1α and GLUT-1 [16]. To the best of our knowledge, there is no previous report of these hypoxic markers in NEC.

In the present study, we retrospectively investigated the clinicopathological features of nasal and laryngeal SCNEC. We also used immunohistochemistry to determine the expression of HIF-1α, GLUT-1, PI3K, and p-Akt protein in these SCNEC.

## Methods

### Patients

The subjects were 10 consecutive patients at The First Affiliated Hospital with histologically demonstrated sinonasal and laryngeal SCNEC between 2003 and 2012, and 15 sinonasal and laryngeal precancerous lesions were also obtained as a control group. Data were obtained from the hospital surgical pathology files.

Our study was approved by the Institutional Review Board of The First Affiliated Hospital, College of Medicine, Zhejiang University. Written informed consent was obtained from each patient before inclusion in the study.

### Immunohistochemistry

Formalin-fixed and paraffin wax-embedded tissue blocks from primary lesions were cut into 4-μm sections, and representative sections were analyzed using immunohistochemistry (EliVision Plus IHC kit; Fuzhou Maixin Biotechnology Development Co., Ltd., Fuzhou, China), using Ki-67 (mouse, clone MIB 1, DAKO, Glostrup, Denmark) at a dilution of 1:400, a rabbit polyclonal antibody against GLUT-1 (1:50, Santa Cruz Biotechnology, Dallas, USA.), a mouse monoclonal antibody against HIF-1α (1:100; Santa Cruz Biotechnology Dallas, USA.), a rabbit monoclonal antibody against PI3K (1:100, Santa Cruz Biotechnology Dallas, USA.), and a rabbit polyclonal antibody against p-AKT (1:50, Santa Cruz Biotechnology, Dallas, USA.). Briefly, the sections were deparaffinized with xylene and dehydrated through an ethanol series. Then, antigen retrieval was performed with a microwave oven over two 10-min cycles. Endogenous peroxidase activity was blocked by incubating the slidesin 1.5% hydrogen peroxide in absolute methanol at room temperature for 10 minutes. Primary antibodies were applied for 1 hour at room temperature, followed by 50 μL of polymer enhancer for 20 minutes and 50 μL of polymerized horseradish peroxidase-anti-mouse immunoglobulin G (IgG) (DAB Kit; Maixin Biological Company,FuZhou, City, China) for 30 minutes. The reaction products were visualized using 3,3’-Diaminobenzidine (DAB Kit; Maixin Biological Company, FuZhou, City, China), and the sections were counterstained with hematoxylin and eosin, dehydrated, and examined under a light microscope. Tris-buffered saline was used in place of the primary antibody for negative controls.

Ki-67, GLUT-1, HIF-1α, PI3K, and p-Akt levels were evaluated by the same investigator (QH You), who was blinded tothe clinicaland follow-up data. GLUT-1 expression was considered positive only if distinct membrane staining was present. HIF-1α, PI3K, and p-AKT proteins were observed in the nucleus and cytoplasm. Protein analysis was performed in 10 random high-power fields,with 100 tumor cells counted within each field for each case and for all antibodies. The percentage of positive cells was calculated by dividing the number of positive tumor cells by the total number of tumor cells counted. A sample was considered negative if less than 25% of the cells were stained.

### Follow-up

The patients were scheduled for follow-up visits every three months after the initial surgery. Follow-up consisted of a routine physical examination and a computed tomography (CT) or magnetic resonance imaging (MRI) scan of the primary site. Patient follow-up was reported up to the date they last seen in the clinic.

### Statistical analysis

The SPSS software (version 20 for Windows; SPSS Inc., Chicago, Illinois, United States) was used to conduct all statistical tests. Associations among GLUT-1, HIF-1α, PI3K, and p-Akt protein expression and pretreatment clinical parameters were analyzed using the chi-squared and Fisher’s exact tests. Overall survival (OS), defined as the time from surgery until death from any cause, was plotted as a Kaplan-Meier curve. Univariate survival analysis was performed using a log-rank test, and multivariate analysis was performed using Cox proportional-hazards regression analysis. A *P *value of less than 0.05 was deemed to indicate statistical significance. The correlation analysis was performed using Spearman’s rank correlation.

## Results and discussion

### Clinicopathological findings

Clinicopathological findings are shown in Table 1. The subjects included eight males and two females with a mean age of 60.8 (range: 53 to71) years. Tumors were located in the maxillary sinus (n = 3, 30%) and the larynx (n = 7, 70%). In the larynx, five cases were located in the supraglottic area and two in the subglottic area. No patient presented with paraneoplastic endocrine syndrome.

**Table 1:** Clinicopathological findings and expression of hypoxic markers

**pt**	**Sex**	**Age (years)**	**Patient**	**Site**	**Clinical features and smoking history**	**Duration of presenting symptom**	**TNM**	**Treatment**	**Recurrence**	**Metastasis**	**Follow-up**	**GLUT-1**	**HIF-1**	**p-Akt**	**PI3K**
1	M	64	1	right nasal cavity and sinuses	right nasal obstruction and bleeding, no smoking	2 months	T2N0M0	right partial maxillectomy and postoperative radiotherapy	yes	No	DOD 22 months	pos	neg	pos	neg
2	M	56	2	left nasal and maxillary sinus	left nasal obstruction and left face numb, 20 cigarettes perday for over 40 years	1 month	T4N0M0	CCRT	yes	yes	DOD 22 months	pos	neg	neg	neg
3	M	50	3	supraglottis	odynophagia, hoarseness,40 cigarettes per day for over 15 years	2 months	T2N0M0	partial laryngectomy, postoperative CCRT	no	no	alive 26 months	pos	pos	pos	neg
4	F	71	4	subglottis	hoarseness, no smoking	8 years	T2N0M0	total laryngectomy	no	no	alive 22 months	pos	pos	neg	pos
5	M	67	5	supraglottis	a progressively enlarging mass in the right side of the neck,20 cigarettes per day for over 20 years	1 month	T_2_N_2_M_0_	total laryngectomy and bilateral neck dissection,postoperative CCRT	no	yes	DOD 12 months	pos	neg	neg	pos
6	M	59	6	supraglottis	odynophagia, no smoking	2 years	T1N0M0	partial laryngectomy, postoperativeCCRT	yes	yes	DOD 18 months	pos	pos	pos	neg
7	M	58	7	subglottis	progressive hoarseness ,40 cigarettes per day for over 20 years	3 months	T2N2M0	total laryngectomy + right neck dissection	no	yes	DOD 6 months	neg	neg	neg	neg
8	M	53	8	supraglottis	hoarseness, 20 cigarettes perday for over 30 years	2 months	T2N0M0	partial laryngectomy, postoperative CCRT	no	no	alive 8 months	neg	neg	neg	pos
9	M	66	9	supraglottis	odynophagia, foreign body feeling, 20 cigarettes per day for over 20 years	2 years	T2N0M0	partial laryngectomy + bilateral neck dissection, postoperative CCRT	no	no	alive 1 months	pos	pos	pos	neg
10	F	64	10	right nasal and ethmoid sinus	left nasal obstruction, no smoking	12 months	T2N0M0	CCRT	yes	yes	DOD 20 months	pos	pos	neg	pos

### Radiological images

**Figure 1:**
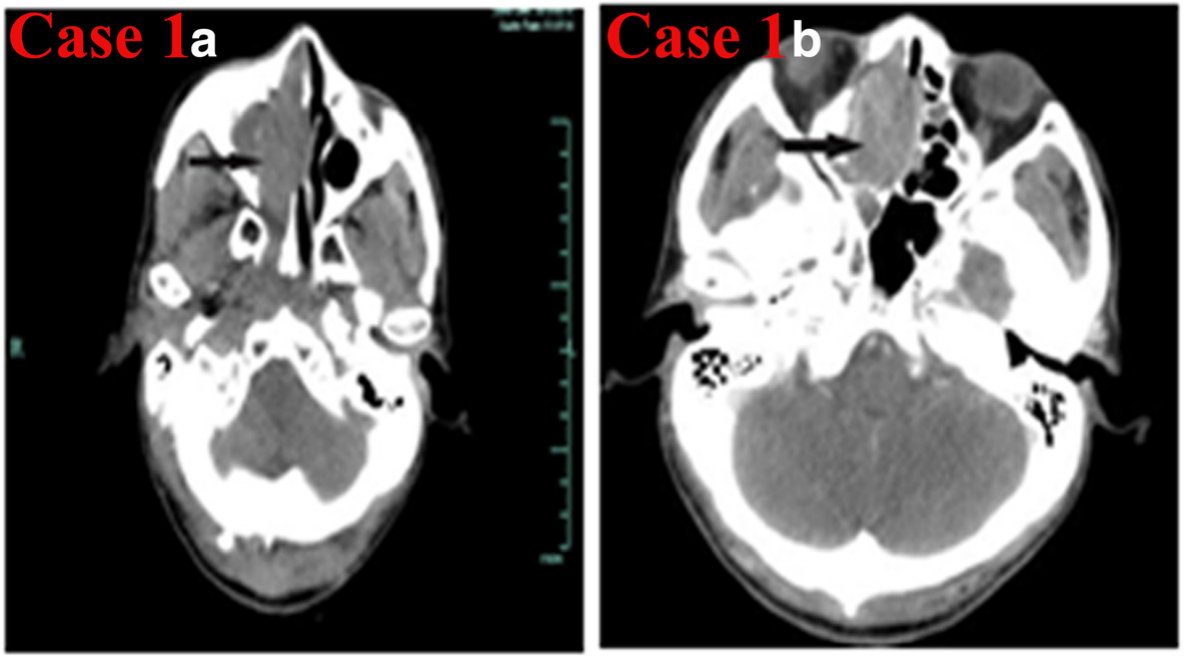
**CT of the sinonasal small-cell neuroendocrine carcinoma of Case 1. a)** Noncontrast axial CT showing a soft-tissue mass in the right maxillary sinus involving the right nasal cavity, right ethmoid sinus, and sphenoid sinus. **b)** Contrast-enhanced CT showing that the lesion was strongly enhanced.

**Figure 2:**
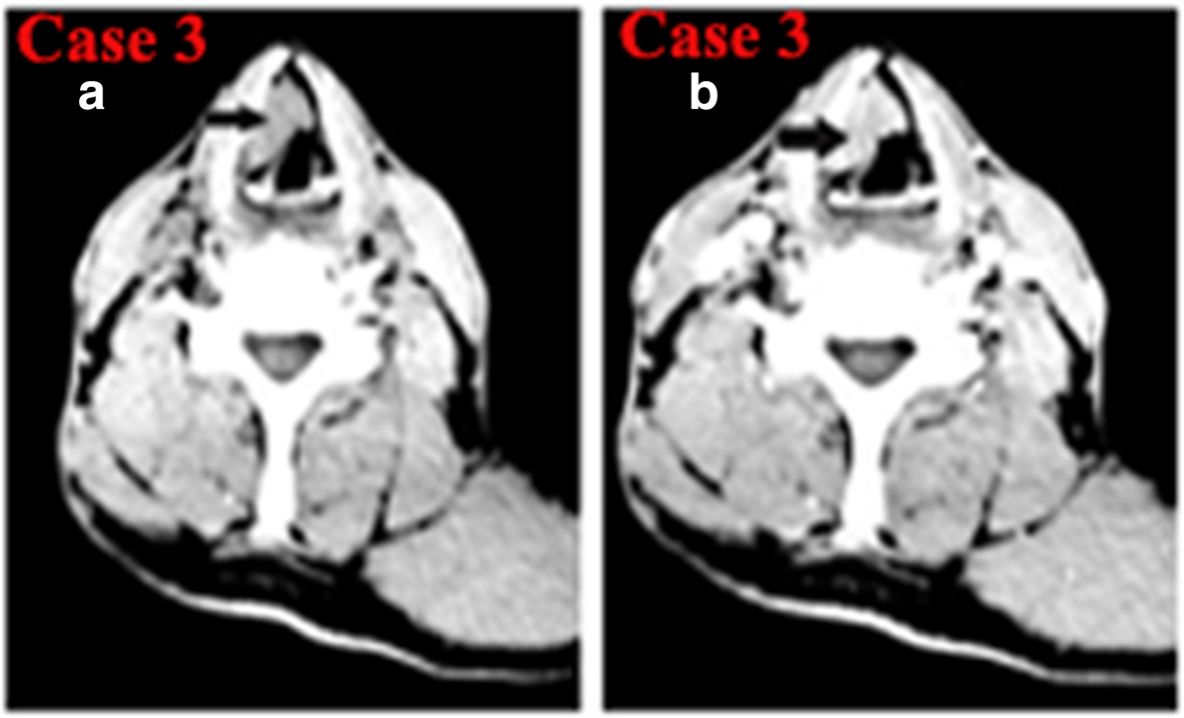
**CT of laryngeal small-cell neuroendocrine carcinoma of Case 3. a)** Noncontrast axial CT showing a heterogeneous irregular soft-tissue mass in the right supraglottic region involving the right vocal cord. **b)** Contrast-enhanced CT showingthat the lesion was heterogeneous enhanced and the thyroid cartilage was not involved.

**Figure 3:**
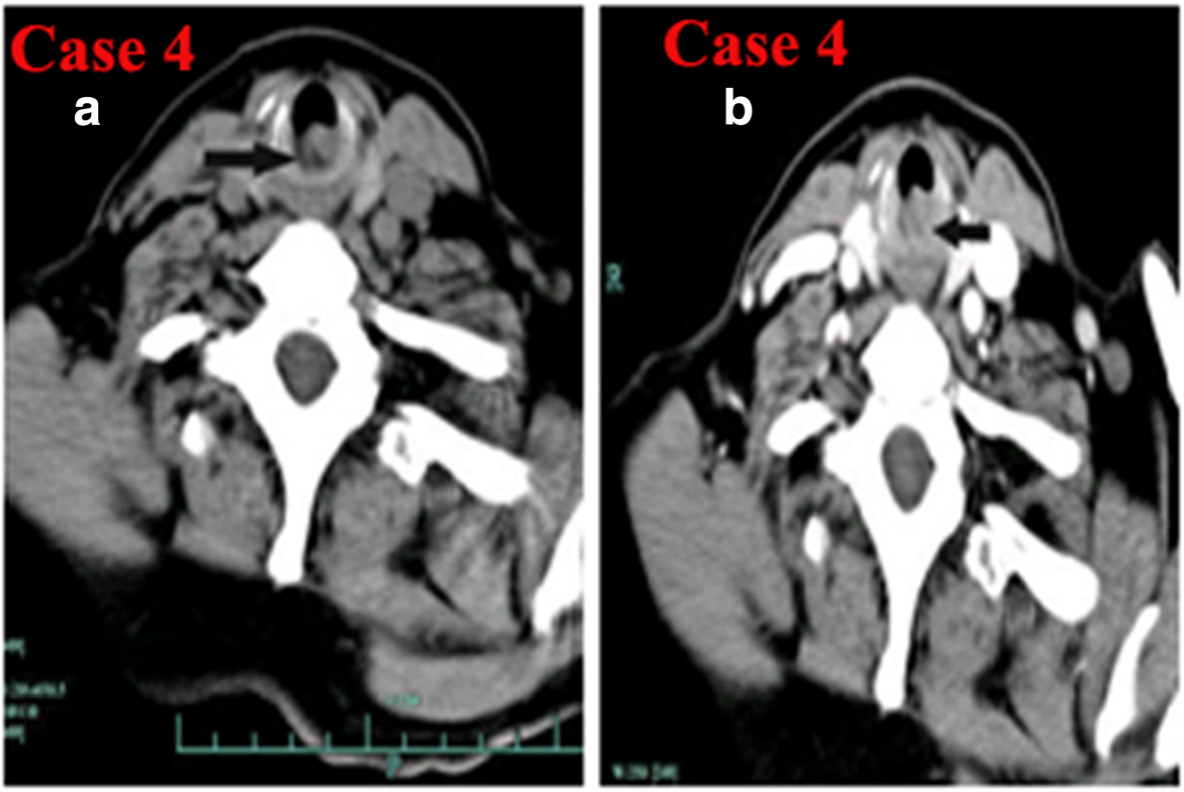
**CT of laryngeal small-cell neuroendocrine carcinoma of Case 4. a)** Noncontrast axial CT showing a heterogeneous well-defined soft-tissue mass in the subglottic regionnot involving the bilateral vocal cord and laryngeal cartilages. **b)** Contrast-enhanced CT showing that the lesion was mildly enhanced.

**Figure 4:**
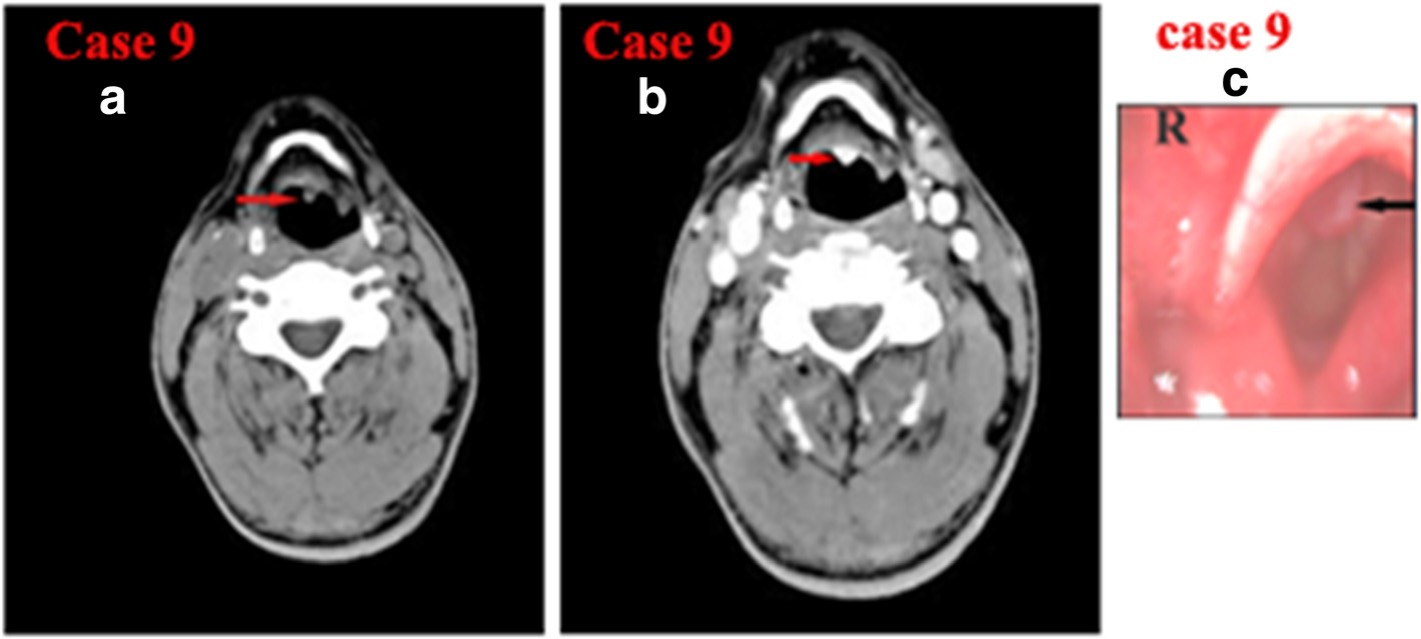
**CT of laryngeal small-cell neuroendocrine carcinoma of Case 9. a)** Noncontrast axial CT showing a small well-defined soft-tissue mass in the left epiglottic base not involving the bilateral vocal cord and laryngeal cartilages. **b)** Contrast-enhanced CT showing that the lesion was strongly enhanced. **c)** Laryngostroscopy showing a red smooth mass in the laryngeal surface of epiglottis.

**Figure 5:**
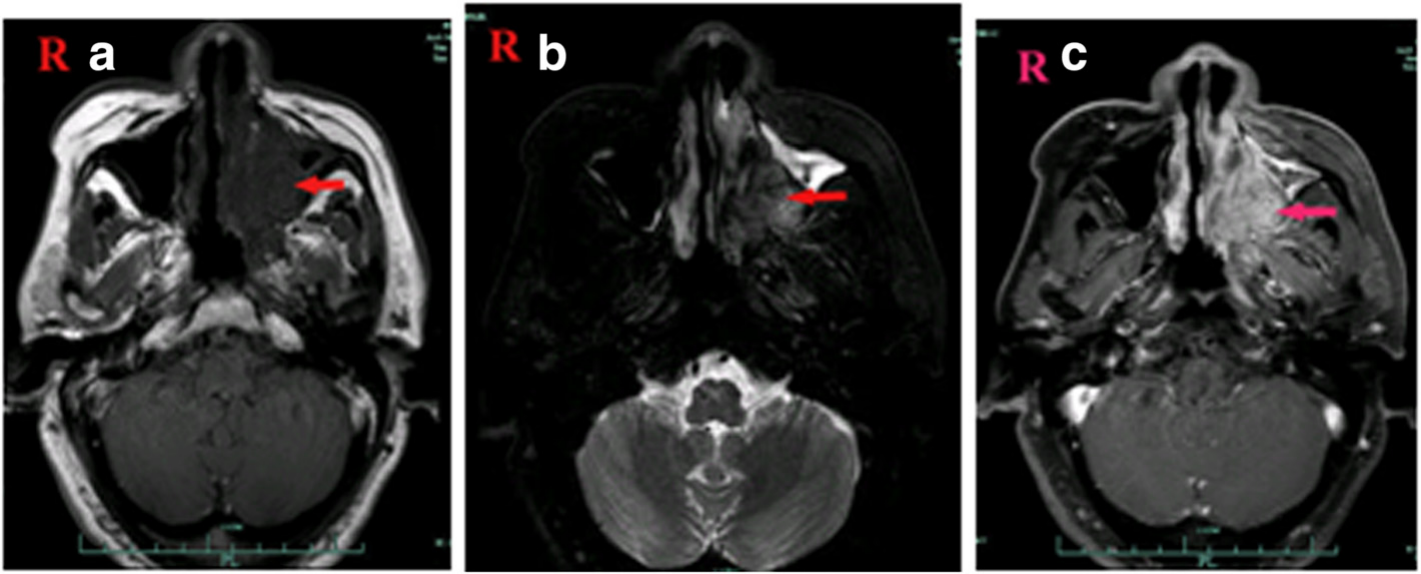
**MRI of Case 2 showingan abnormal signal in the left maxillary sinus, left nasal cavity, and nasopharynx. a)** TheT1-weighted signals were hypointense, **b)** the T2-weighted signals were hyperintense, **c)** and the contrast-enhanced T1-weighted MRI images show strong enhancement.

**Figure 6:**
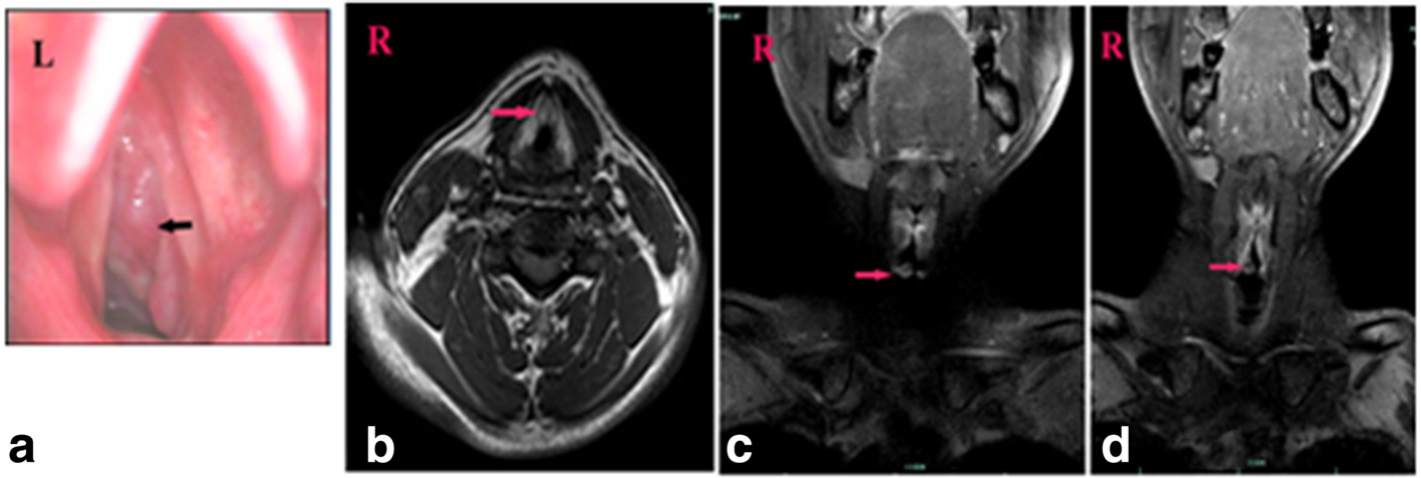
**Laryngostroscopy and MRI of Case 7. a)** Laryngostroscopy showed that there was a red smooth mass in the right subglottic area. An MRI scan revealed that there was a mass in the right subglottic area. **b)** TheT1-weighted signals, **c)** and the T2-weighted signals were hyperintense,**d)** and the contrast-enhanced T1-weighted MRI images show strong enhancement.

**Figure 7:**
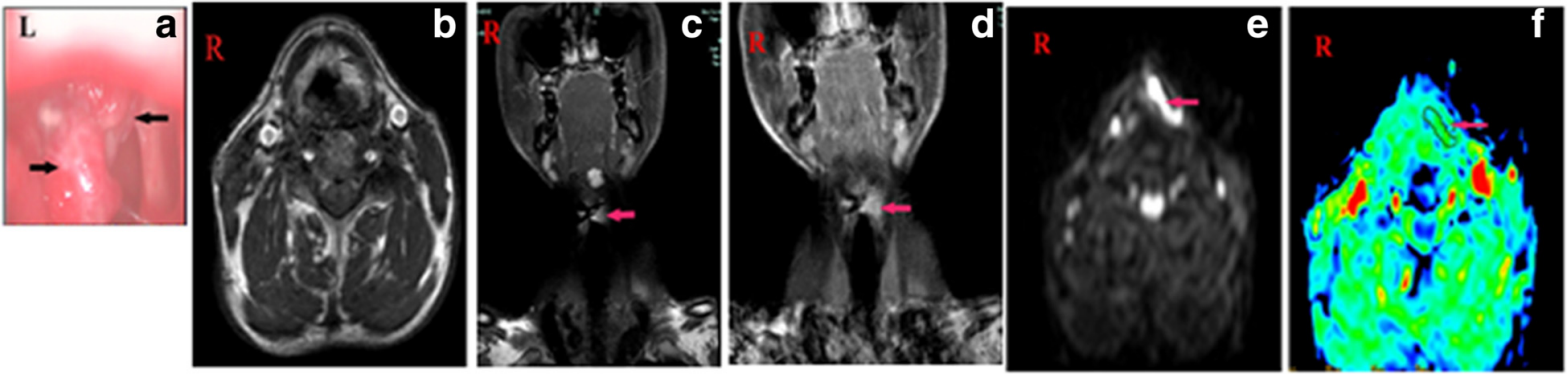
**Laryngostroscopy and MRI of Case 8. a)** Laryngostroscopy showinga red smooth mass in the left false vocal cord involving in the left vocal cord, anterior commission, and left aryepiglottic fold. An MRI scan revealed that there was a mass in the left false vocal cord that also involved the left vocal cord. **b)** TheT1-weighted signals and **c)** the T2-weighted signals were hyperintense, **d)** and the contrast-enhanced T1-weighted MRI images show strong enhancement. **e)** DWI suggested hyperintense lesions were in the left false vocal cord (b = 1000s/mm^2^). **f)** The corresponding ADC map reveals a hypointense mass of lesions in the left false vocal cord (ADC = 0.793 × 10^-3^ mm^2^/s).

**Figure 8:**
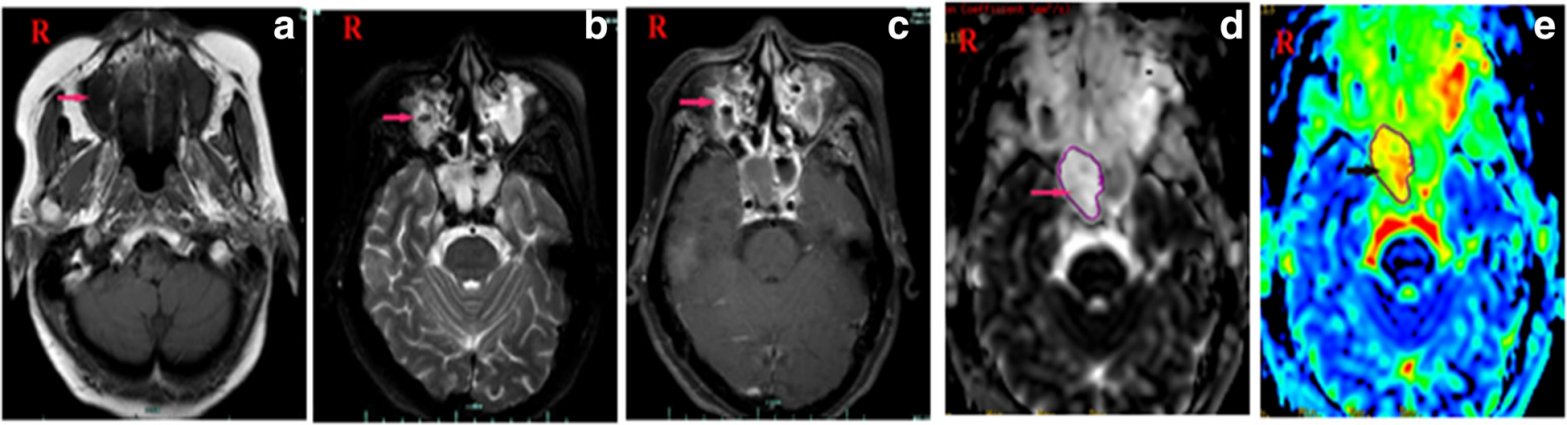
**MRI of Case 10 showingan abnormal signal in the right maxillary sinus and right nasal cavity. a)** TheT1-weighted signals **b)** and the T2-weighted signals were hyperintense **c)** and the contrast-enhanced T1-weighted MRI images show strong enhancement.** d)** DWI suggested hyperintense lesions were in the right maxillary sinus and right nasal cavity. **e)** The corresponding ADC map reveals (ADC = 1.78 × 10^-3^ mm^2^/s).

The SCNEC radiological images available varied. CT images were available for eight patients and showed homogenous (two cases) and heterogeneous (six cases) soft-tissue masses. Contrast-enhanced CT images were available for eight patients: mild enhancement was found in the two cases of laryngeal SCNEC and strong enhancement in six cases (one in the nasal cavity, five in the larynx; Figures 1, 2, 3 and 4). MRI data were available for four patients (two in the nasal cavity and two in the larynx). In four patients, the T1-weighted signals and the T2-weighted signals were hyperintense. Contrast-enhanced T1-weighted MRI images showed strong enhancement in all four patients. Diffusion-weighted MRI (DWI) was available for two patients. The DWI in two patients was high-signal and the apparent diffusion coefficient (ADC) was 0.793 ± 0.33 × 10^-3^ mm^2^/s (laryngeal SCNEC) and 1.78 ± 0.16 × 10^-3^ mm^2^/s, respectively (Figures 5, 6, 7 and 8).

### Treatment and follow-up

In nasal SCNEC, two patients received concurrent chemo-radiotherapy (CCRT) and one patient received partial maxillectomy and postoperative radiotherapy. Three patients developed local recurrence and two patients developed distant metastasis. Three patients died at 22, 22, and 20 months after diagnosis. In larynx SCNEC, four patients underwent horizontal supraglottic laryngectomy and one patient received bilateral neck dissection simultaneously. All four patients received postoperative CCRT. One patient was alive at over 26 months. Two patients were followed for only 1 and 6 months after surgery. One patient suffered a local recurrence 14 months after initial surgery and underwent a total laryngectomy and bilateral neck dissection and postoperative radiotherapy. The patient died 18 months after the first surgery due to distant metastasis. Three patients underwent total laryngectomies. One patient received bilateral neck dissection and postoperative CCRT. He died 12 months after surgery. One received a unilateral neck dissection and died 7 months after diagnosis due to a distant metastasis. Another patient received 14-Gy postoperative radiotherapy. However, she did could not tolerate the side effects of radiotherapy and abandoned it. She was disease-free after 22 months of follow-up. In this small series, the mean overall survival (OS) was 19.3 ± 2.1 months and the two-year survival rate was 25.4%.

### Immunohistochemical findings

**Figure 9:**
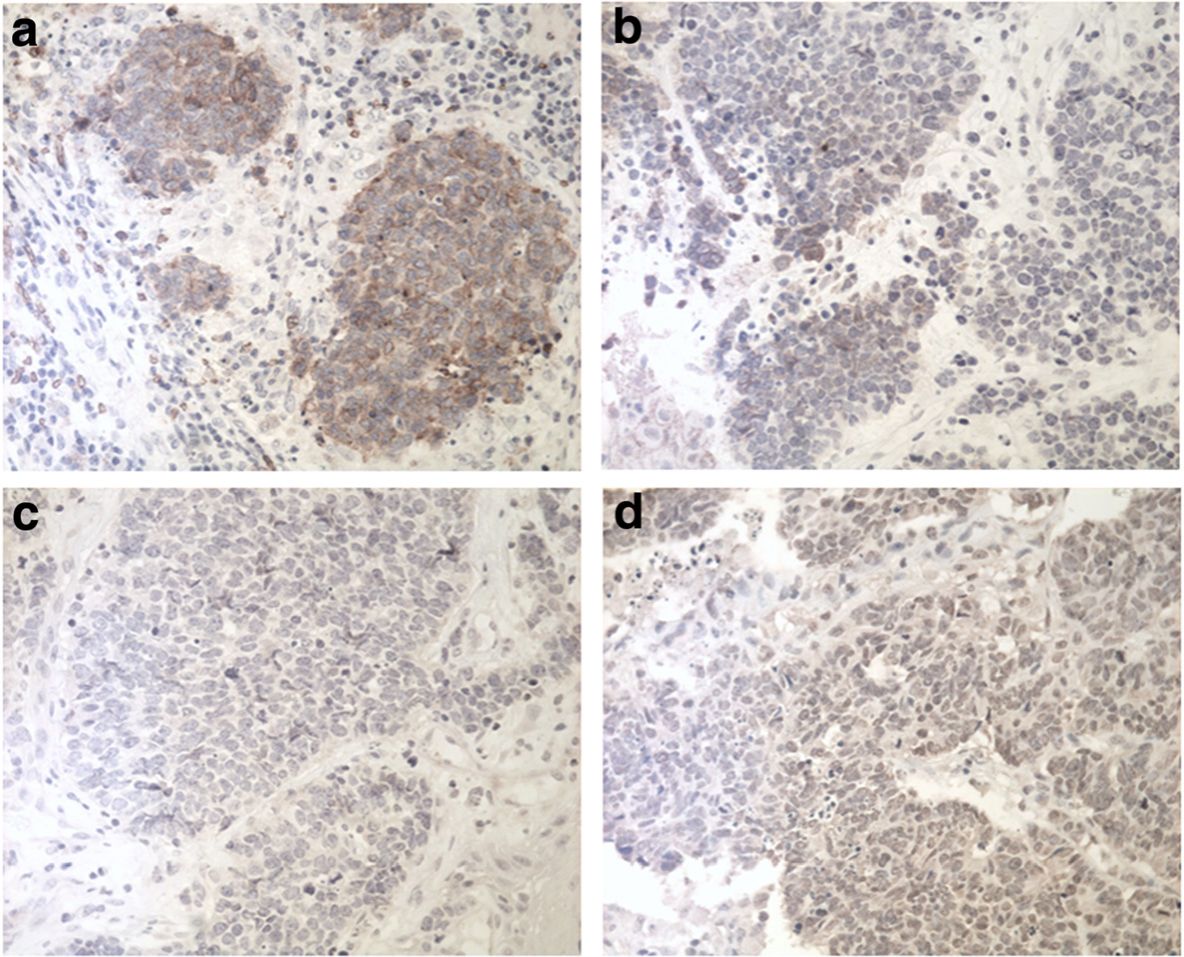
**The expression of hypoxic markers in the sinonasal and laryngeal small-cell neuroendocrine carcinoma.** The expression of **a)** GLUT-1, **b)** HIF-1α, **c)** PI3K, and **d)** p-Akt was positive.

The Ki-67 index was 25% in one patient, 30% in two patients, 45% in three patients and higher than 70% in four.The cutoff of the Ki-67 for the diagnosis of small-cell carcinomaswas 50%. Expression of GLUT-1, HIF-1α, PI3K, and p-Akt was seen in sinonasal and laryngeal SCNEC in 80 (8 out of 10), 50 (5 out of 10), 40 (4 out of 10), and 40% (4 out of 10) of cases, respectively (Table 1, Figure 9). Expression of GLUT-1, HIF-1α, PI3K, and p-Akt in control precancerous lesions was seen in 6.7 (1 out of 15), 0 (0 out of 15), 0 (0 out of 15), and 0% (0 out of 15) cases, respectively. The expression of GLUT-1, HIF-1α, PI3K, and p-Akt was higher in sinonasal and laryngeal SCNEC than in precancerous lesions (*P* = 0.001, 0.005, 0.017, and 0.017, respectively). Of the five patients who died, five (100%) were positive for GLUT-1 and two (40%) were positive for HIF-1α, PI3K, and p-Akt.

In this small series, univariate analysis showed that poor survival was significantly associated with distant metastasis (χ^2^ = 4.97, *P* = 0.026). Ki-67, GLUT-1, HIF-1α, PI3K, and p-Akt expression were not correlated with survival.In a multivariate analysis,these markers were not predictors of OS.

Sinonasal and laryngeal SCNEC typically presents therapeutic challenges, and specific etiological factors and prognostic factors remain unknown due their extreme rarity. Nasal and laryngeal SCNEC is the most aggressive and has a poor prognosis. Unlike other reports of sinonasal NEC, which is most common in the ethmoid sinuses [3], in our series, all three sinonasal tumors were located in the maxillary sinus. In the larynx, five cases were located in the supraglottic area and two in the subglottic area. There was a male predominance, with a male/female ratio 4:1, and a mean age of 60.8 (range: 53 to 71) years. Seven patients were heavy smokers. The mean duration of symptoms at presentation was 16.7 months (range: 1 month to 8 years). In another report of 16 cases of NEC in the head and neck, the male/female ratio was 1.7:1, and the mean age was 65.8 (range: 43 to 88) years. Of those, 11 (68.8%) had smoked cigarettes (mean pack-years: 50.5; range: 15 to 116) [17].

CT or MRI imaging of the head and neck are more useful than conventional radiography when assessing the extent of the local invasion of the tumor and are better for the planning of further treatment [6]. In our series, CT scans showed heterogeneous soft-tissue masses in most cases and a mass in the right maxillary sinus extending to the right nasal cavity in one case. Contrast-enhanced CT images showed that the lesions had differing degrees of enhancement. An MRI scan improves differentiation between inflammatory reaction, tumor, and liquid retention. The combination of DWI and ADC may differentiate malignant tumors from precancerous lesions and benign tumors [18]. In the present study, the T1-weighted signals and the T2-weighted signals were hyperintense. Indeed, contrast-enhanced T1-weighted MRI images showed a strong enhancement. We first reported DWI and ADC values in head and neck SCNEC. We found high DWI and low ADC values in laryngeal SCNEC compared with our previous findings [18]. Future studies with larger patient populations are recommended to further evaluate the role of DWI in head and neck NEC.

The treatment of sinonasal and laryngeal SCNEC is controversial. Surgery, radiotherapy, and chemotherapy alone, or in combination, have been used for sinonasal SCNEC [16]. In the present study, two of the three patients with sinonasal SCNEC received CCRT and one patient received surgery and postoperative radiotherapy. Three patients died within two years.In the 1980s, surgery followed by radiotherapy was the routine therapeutic strategy for sinonasal SCNEC [19]. Since the late 1990s, a combination of chemotherapy and radiotherapy, with or without surgery, has been recommended [6]. Regarding the treatment of laryngeal SCNEC, most authors generally agree that surgery, alone or in combination with radiotherapy, does not improve local tumor control [2]. Baugh *et al.* found that the combination of primary radiation therapy and adjuvant chemotherapy resulted in relative good prognosis through comparison of various previously reported therapeutic modalities for laryngeal SCNEC [20]. However, this review was published in 1986 and concerned approximately 50 cases. Since then, there has been no large series report of laryngeal SCNEC and there is no review or meta-analysis of the treatment of laryngeal SCNEC. Thus, no standard therapeutic modality has yet been determined for laryngeal SCNEC. In the present study, four patients underwent partial laryngectomies plus postoperative CCRT and three patients underwent total laryngectomy. Two patients died of distant metastases. The other six patients were alive, but the follow-up duration is insufficient.

Extra-pulmonary small-cell carcinoma has a dismal prognosis, with a 13% five-year survival rate [4]. The prognosis of sinonasal and laryngeal SCNEC is poor and is similar to that of small-cell lung cancer (SCLC). In larynx SCNEC, the two-year survival rate was 16%, while the five-year rate was only 5% [5]. In the nasal cavity and sinus, the five-year overall survival for SCNEC is below 30% [6]. However, the prognostic factors remain unclear. Unfavorable prognostic factors of SCNEC in the head and neck seem to be correlated with an invasion of the lamina cribrosa [8], recurrence, and distant metastasis [5]. In pulmonary SCNEC, ectopic hormone syndrome may be a predictor of increased mortality due to the higher risk of cerebral metastasis [21]. Endocrine syndrome also seems to worsen the prognosis in cases of head and neck SNEC [6]. Almost 50% of the patients with laryngeal SCNEC have positive lymph nodes at the initial diagnosis and 90% of them develop distant metastases (for example lymph nodes, liver, lung, bones, or bone marrow) [22]. In the present laryngeal SCNECs, only two patients (28.6%) had positive lymph nodes at the time of symptom presentation. At the final follow-up, only one patient (14.3%) had local recurrence and three patients (42.9%) developed distant metastases, including in the liver, lung, and cervical lymph node. Unlike laryngeal SCNEC, patients with sinonasal SCNEC had low lymph node metastatic rates at symptom presentation. Babin *et al.* reported that only three patients (14.3%) had positive lymph nodes at the initial diagnosis [6]. In their series, 17 of 21 patients suffered relapses or metastases within the first two years [6]. Han *et al.* reviewed 55 cases of sinonasal SCNEC in the English literature [3]. They found that the overall local recurrence rate was 33% and the metastasis rate was 31% [3]. In 11 patients with head and neck SCNEC, Meacham *et al.* found that eight patients had regional lymph node metastases and another patient had distant metastases. Of patients with SCNEC in the head and neck, 35.4% survived to 24 months [19]. In our series, three patients had no metastatic cervical lymph nodes at initial diagnosis. In our small series, the mean overall survival was 21.3 months. A high Ki-67 index is an unfavorable sign in some NECs [23]. An unfavorable course is observed when the Ki-67 index is higher than 5% [24]. In our study, the Ki-67 index was higher than 25% in eight patients.However, univariate analysis showed that poor survival was not associated with Ki-67.

Overexpression of HIF-1αis considered to be a significant poor prognostic factor in SCLC as well as non-SCLC [9,10]. In SCLC, low expression of HIF-1α may be a useful predictor of better overall survival [10]. HIF-1α expression had an unfavorable influence on overall survival in SCLC [9]. HIF-1α upregulates expression of GLUT-1 via the PI3K/Akt pathway [16]. GLUT-1 was expressed in approximately half of the pulmonary NEC, and GLUT-1 expression was associated with an increased risk of death in pulmonary NEC [11]. Although there was no significant difference between HIF-1α/GLUT-1/PI3K/Akt expression and sinonasal and laryngeal SCNEC, this is the first study of HIF-1α, GLUT-1, PI3K, and p-Akt expression in head and neck NEC. In this study, we found that HIF-1α, GLUT-1, PI3K, and p-Akt expression was significant higher in sinonasal and laryngeal SCNEC than in precancerous lesions. Of the five patients who died, five (100%) were positive for GLUT-1 and two (40%) were positive for HIF-1α, PI3K, and p-Akt. Because of the small number of cases in our series (n = 10), it is difficult to reach any statistically significant conclusions regarding the prognostic significance of these hypoxic markers. The effect of the expression of these hypoxic markers on the prognosis in sinonasal and laryngeal SCNEC requires further clarification with a larger cohort.

## Conclusions

Primary sinonasal and laryngeal SCNEC is rare. In our case series, laryngeal SCNEC was more common. Sinonasal and laryngeal SCNEC present with common local recurrence and distant metastasis. CT and MRI scans may aid in the diagnosis and assessment of the extent of local invasion. The mean overall survival was 19.3 months in 10 sinonasal and laryngeal SCNEC cases.The best treatment of sinonasal and laryngeal SCNEC is still unclear. HIF-1α, GLUT-1, PI3K, and p-Akt expression was significant higher in sinonasal and laryngeal SCNEC than insinonasal and laryngeal precancerous lesions. An examination of a larger series is required to identify prognostic factors and formulate appropriate therapeutic strategies.

## Abbreviations

ADC: Apparent diffusion coefficient; CCRT: Concurrent chemo-radiotherapy; DWI: Diffusion-weighted MRI; EGFR: Epidermal growth factor receptor; F-FDG: 18 F-2-fluro-2-deoxy-d-glucose; GLUT-1: Glucose transporter-1; HIF-1α: Hypoxia-inducible factor 1α; NEC: Neuroendocrine carcinoma; PI3K/Akt: Phosphatidylinositol 3-kinase/protein kinase B pathway; SCLC: Small-cell lung cancer; SCNEC: Small-cell neuroendocrine carcinoma; VEGF: Vascular endothelial growth factor.

## Competing interests

The authors declare that they have no competing interests.

## Authors’ contributions

LC participated in study design and aided surgeries. S-HZ conceived and designed the study, performed surgery, participated in data collection, analyzed the data, and drafted the manuscript. H-TY contributed to the study design andperformed immunohistochemistry. Q-HY evaluated the results of immunohistochemistry. H-FY, T-TW and Y-YB collected the materials and follow-up. All authors read and approved the final manuscript.
